# Academic Coaching to Promote Self-Directed Learning in Graduate Medical Education

**DOI:** 10.1007/s11606-025-09424-7

**Published:** 2025-02-13

**Authors:** Kathryn M. Burtson, Kelsey R. Wilson, Michelle E. Kiger, Eulho Jung, Joshua D. Hartzell, Holly Meyer

**Affiliations:** 1 Wright Patterson Medical Center, Wright Patterson AFB, OH USA; 2 Department of Internal Medicine, Wright State University Boonshoft School of Medicine, Dayton, OH USA; 3 Department of Health Profession Education, Uniformed Services University, Bethesda, MD USA; 4 Department of Pediatrics, Wright State University Boonshoft School of Medicine, Dayton, OH USA

**Keywords:** academic coaching, self-directed learning, mixed-methods, case study, graduate medical education

## Abstract

**Background:**

Graduate medical education is a critical period for fostering self-directed learning (SDL). This study introduced an academic coaching program to support SDL among internal medicine (IM) residents, leveraging Gallimore and Tharp’s four-stage model as a scaffolding framework.

**Objective:**

To assess the impact of academic coaching on residents’ performance, including Internal Medicine In-Training Examination (IM-ITE) scores, individualized learning plans (ILPs), and attitudinal changes. The study also explored how coaching influenced SDL within the residency program.

**Design and Participants:**

A mixed-methods case study was conducted in a mid-sized university’s IM residency program. Quantitative measures included pre- and post-coaching surveys, ILP analysis, and IM-ITE score evaluation. Semi-structured interviews provided qualitative insights into participant experiences. Of 77 eligible residents, 40 enrolled in the coaching program, and 27 (18 post-graduate year (PGY) 1 and PGY2) completed at least one session. Baseline IM-ITE scores guided enrollment for mandatory participants.

**Key Results:**

Of the 77 residents, 51 had complete IM-ITE data and individualized learning plans (ILPs) from 2022 and 2023. Residents attending one coaching session demonstrated significant improvement in IM-ITE percentile scores (*p* = .022), while those with two or more sessions showed significant gains in both percent correct (*p* = .015) and percentile scores (*p* = .003). No significant differences were observed in ILPs or attitudinal surveys. Qualitative analyses of resident participant interviews highlight coaching’s positive influence on SDL, organized into input, process, and output domains.

**Conclusions:**

Sustained coaching, defined as two or more coaching meetings, is associated with improved IM-ITE performance. Qualitative findings underscore the program’s role in enhancing residents’ SDL.

**Supplementary Information:**

The online version contains supplementary material available at 10.1007/s11606-025-09424-7.

## INTRODUCTION

In 2020, the doubling time of medical knowledge was projected to be just 73 days—far faster than our ability to assimilate new information.^1^ The pace of updates and breadth of medical knowledge call for fundamental change in how educators prepare GME graduates for a future of lifelong self-directed learning (SDL).^1–3^

As GME emphasizes SDL, coaching will be essential to guide residents in obtaining this skill by providing the needed scaffolding.^3–5^ Based on Vygotsky’s concept of the zone of proximal development (ZPD), which describes the gap between what a learner can do independently and what they can achieve with guidance and support from a more knowledgeable individual, scaffolding involves structured support from an educator that helps trainees progress beyond their current abilities to achieve higher levels of understanding and independence.^6,7^ Although coaching programs are still gaining traction within GME platforms, research indicates that they empower residents by helping them develop goals, identify gaps between their current and ideal states, and create actionable plans.^8–14^ Academic coaching, a specific coaching archetype, is a process led by a dedicated individual who supports and guides learners in reaching their fullest potential.^11^

With residents’ increasingly formal expectations for engaging in SDL, we require systematic knowledge about how a coach can support this process. No studies have examined the influence of academic coaching on SDL. Most existing studies on coaching within GME have been quantitative and focused on coaching for well-being, facilitated transitions, and non-technical and technical skills.^14,15^ Quantitative approaches are less suited to explore how coaching influences SDL, and monomethod approaches present validity risks secondary to common method variance.^16^ Understanding the mechanisms by which coaching can impact SDL will allow educators to design and implement coaching programs to optimize the learning trajectory for residents in training and beyond. Therefore, we employed a mixed-methods case study approach to examine the impact of a coaching program within an IM residency program to gain a deeper understanding of the connections between coaching and SDL. We employed a theory-informed inductive study design based on Gallimore and Tharp’s four stages. Rooted in Vygotsky’s ZPD, Gallimore and Tharp’s four-stage model delineates a process by which trainees’ degree of scaffolding and support gradually decreases as they gain proficiency and move towards independent practice. Over time, the support becomes more subtle, focusing on refining skills and fostering self-regulation until they eventually achieve automaticity and can perform tasks independently without external aid.^17^ Using this conceptual model as a basis for our coaching program, we asked, “How does coaching impact residents’ SDL within an IM residency program?”

## METHODS

### Sample and Setting

This study spanned 12 months from 2022 to 2023, using the September Internal Medicine In-Training Examinations (ITEs) as the starting and ending points of the program. Informed by the American Medical Association Coaching Workshop, KB and KW created resident and faculty workshops to introduce a coaching program focused on improving residents’ SDL. This workshop introduced coaching and its unique role in GME. As formative exercises, the residents critiqued and developed SMART goals. All 77 residents used their IM-ITE results to create individualized learning plans (ILP) in SMART goal format for the academic year (Appendix a).^18^ A total of 40 residents enrolled in the coaching program, comprising both mandatory and elective participants from post-graduate years (PGY) 1, 2, and 3. Notably, the mandatory participants were those who scored below the 30th percentile on the IM-ITE.

The academic coaches comprised 13 faculty volunteers from three hospital sites and four specialties (IM, endocrinology, rheumatology, and pulmonary critical care). Coaches attended a 1-h faculty development session and were provided with coaching frameworks, scripts, and contracts (Appendixes b, c, d). KW organized coach assignments to ensure coaches were not residents’ advisors, preserving role integrity. Coaches were randomly assigned three or four residents, with meetings held separately from the sessions of the advisor or program director. The expectation was set for bimonthly meetings between the coach and coachee. Program leadership sent monthly check-in emails to the academic coaches.

### Data Collection and Analysis

#### Survey

We developed an attitudinal survey on resident perceptions of coaching and SDL and refined the questions using cognitive interviews with a think-aloud approach.^19^ We pilot-tested the interview questions on two IM chief residents. The pre-survey was distributed at the program’s start, while the post-survey was conducted before graduation for PGY3 residents and prior to the 2023 In-Training Examination for PGY1 and PGY2 residents. The 1-to-5 Likert-rating scale responses were analyzed in a controlled pre-test post-test design with a two-tailed paired *t*-test in IBM SPSS Statistics Version 29.0.2.0.

#### IM-ITE Examination Scores (IM-ITE)

Paired data for assessing changes in IM-ITE scores were only available for PGY1 and PGY2 residents form the 2022 cohort, as they had complete pre-coaching and post-coaching data, enabling a longitudinal analysis of score changes. Each outcome’s coached and not-coached cohorts were analyzed in a controlled pre-test post-test design with a two-tailed independent-samples *t*-test in SPSS. The cohorts in the study were not randomized but were determined based on their enrollment in the coaching program. The analysis included only residents with paired data, specifically those who were PGY1 or PGY2 in 2022. This allowed for the assessment of changes in percent correct and percentile between the 2022 and 2023 IM-ITE examinations.

#### Interviews

We conducted virtual semi-structured interviews via Google Meet with residents who had met their assigned coach at least once. Of the 27 eligible participants, 16 were interviewed, with purposeful selection based on coaching engagement. Purposeful sampling was used to select participants involved in the coaching program, with inclusion criteria requiring attendance at least one coaching session. This approach ensured the selection of individuals most likely to provide meaningful insights. Sampling continued until data saturation was reached.

We used an interview guide to direct our interviews (Appendix a). Interviews were digitally recorded and transcribed. KB verified transcripts for accuracy against recordings. The interviews were designed to better understand definitions, experiences, and expectations regarding the relationship between coaching and SDL. We stopped obtaining new interviews when no new ideas were being shared in the interviews, and we believed we had rich data to answer our research question, such that thematic saturation was achieved.

We analyzed data using thematic analysis following Braun and Clarke’s six-step process.^20,21^ After familiarization with the data, we coded transcripts using open and axial coding. Four authors independently coded two transcripts before codes were combined through consensus to develop a code book. Throughout the coding process, authors memoed to capture spontaneous ideas and thoughts about data and refined the coding scheme based on discussion.^22^ For the remaining transcripts, KB performed the first pass of coding, and JH, HM, MK, and KW completed a second pass of coding. The authors employed an inductive approach to searching for initial themes, deriving ideas exclusively from research data. As we began to review and define themes as a group, we utilized a program evaluation logic model and concepts from Gallimore and Tharpe’s four-stage model as sensitizing concepts to organize our themes. To analyze this data, we applied the input-process-output (IPO) model, a classical business framework used to assess team effectiveness.^23^ According to McGrath (1964), individual, group, and environmental variables drive group interactions, affecting performance. We have organized and presented the findings in three distinct domains: (a) input, (b) process, and (c) output. The manuscript was provided to interviewees for member-checking.

#### Mixed Methods

We utilized a case study design. Multiple methods are intended to measure the same phenomena and are designed to be of equal weight. The qualitative semi-structured interviews, quantitative questionnaires, and impact exploration were implemented concurrently with the intention of complementarity. Qualitative methods were selected to assess the quality of the coaching program implementation, quantitative methods were chosen to evaluate the magnitude of program outcomes, and mixing methods were used to help understand the outcome results. These methods differ in important ways for generative potential and the potential to respect, appreciate, and accept variation in our phenomena of interest.^24^

#### Reflexivity

KB is the residency program director and co-created the academic coaching program with KW, her Associate Program Director. Since her primary goal was to assess the program’s effectiveness, the team adopted a pragmatic orientation to data analysis. MK is a general pediatrician and former program director with expertise in qualitative research methodology. HSM is a health education researcher with expertise in coaching and qualitative methods. MK and EJ performed all the interviews for residents who were required to participate in the coaching program to mitigate potential biases and maximize participant comfort during the interviews.

The Wright Patterson Medical Center Human Research Protection Program performed an ethical review of this study, and protocol #FWP20230008N was approved with a “Not Research” determination.

## RESULTS

### Quantitative Results

After five sessions, 76 of 77 Internal Medicine residents (99%) participated in a 2-h interactive coaching workshop, with all residents completing an Individualized Learning Plan (ILP). Residents identified as academically at-risk by scoring less than the 35th percentile on their national IM-ITE 29/77 (38%) were required to enroll in the academic coaching program. All remaining residents were invited to participate, and 11/48 (23%) registered. The coaching program included residents across all postgraduate levels: first, second, and third years (Table 1). Fifty-one residents had IM-ITE outcome data for the 2022 and 2023 academic years. Residents who met with a trained coach at least once had a significant improvement in performance on their IM-ITE percentile score compared to residents who did not partake in the coaching program, *p* = 0.022, ES = 0.691 (Table 2). Furthermore, residents who met with a trained coach at least twice had statistically significant improvements in their IM-ITE percent correct compared to residents who did not participate in the coaching program, *p* = 0.015, ES = 0.788, and percentile score compared to residents who did not participate in the coaching program, *p* = 0.003, ES = 0.999 (Table 2). No significant differences were associated with annotating in individualized learning plans and performance on the IM-ITE (Table 3).

**Table 1:** Engagement Patterns in an Internal Medicine Residency Coaching Program: A PGY-Level Analysis

PGY	Enrollment type	Total registrants	Not coached (0 sessions)	Coached (≥ 1 session)	Coached (1 session)	Coached (≥ 2 sessions)
PGY-1	Mandatory	8	4	4	1	3
PGY-2	Mandatory	11	3	8	0	8
PGY-3	Mandatory	10	3	7	2	5
PGY-1	Elective	4	0	4	1	3
PGY-2	Elective	3	1	2	0	2
PGY-3	Elective	4	2	2	0	2
Total		**40**	**13 (32.5%)**	**27 (67.5%)**	**4 (10%)**	**23 (57.5%)**

**Table 2:** Evaluation of Changes in IM-ITE Scores from 2022 to 2023 Based on Coaching Participation and Individualized Learning Plan Use

Group comparison	*n*	M	SD	df	*t*	*p*	Cohen’s *d*
Percent correct							
Coached	18	7.17	5.03	49	− 1.723	0.091	0.505
Not coached	33	5.00	3.84				
Percentile							
Coached	18	9.83	18.75	49	− 2.357	0.022	0.691
Not coached	33	− 1.30	14.54				
Percent correct							
Met ≥ 2 times	14	8.14	4.24	49	− 2.511	0.015	0.788
Met once or not coached	37	4.86	4.13				
Percentile							
Met ≥ 2 times	14	13.88	17.75	49	− 3.185	0.003	0.999
Met once or not coached	37	− 1.62	14.59				
Percent correct							
ILP annotated	11	6.55	5.34	49	− 0.665	0.509	0.226
ILP not annotated	40	5.55	4.13				
Percentile							
ILP annotated	11	7.09	17.75	49	− 0.992	0.326	0.338
ILP not annotated	40	1.40	16.61

**Table 3:** Differences in Pre- and Post-Intervention Attitudinal Questionnaire Scores Between Coached and Not Coached Residents

	Coached (*n* = 20)		Not coached (*n* = 22)		df	*t*	*p*	Cohen’s *d*
	M	SD	M	SD				
How frequently do you perform self-directed learning?	0.3000	1.03110	0.1818	0.79501	40	− 0.418	0.678	0.129
How satisfied are you with your self-directed learning?	0.5000	1.05131	0.2727	0.98473	40	− 0.723	0.474	0.223
How much effort do you put into your self-directed learning?	0.3000	0.8645	− 0.0909	0.81118	40	− 1.512	0.138	0.467
How important is your self-directed learning to your career progression?	0.2500	0.7864	0.0455	0.72225	40	− 0.879	0.385	0.271
How confident are you in your self-directed learning?	− 0.3000	0.86450	− 0.4545	1.01076	40	− 0.530	0.599	0.164
How important are the creation of SMART goals to your career progression?	− 0.5500	0.68633	− 0.6364	1.17698	40	− 0.287	0.776	0.089
How confident are you in your ability to construct SMART goals?	− 0.1000	0.78807	− 0.5000	0.80178	40	− 1.628	0.111	0.503
How important is having an academic coach for your career progression?	− 0.2500	1.06992	− 0.6818	1.08612	40	− 1.296	0.202	0.400
How confident are you that academic coaching can help you reach your career goals?	− 0.3000	0.86450	− 0.4545	0.85786	40	− 0.581	0.565	0.179
How confident are you in your ability to distinguish between the distinct roles of advisor, mentor, and coach?	− 0.4000	0.59824	− 0.7727	0.97257	40	− 1.478	0.147	0.457

The pre-and post-surveys on coaching were disseminated before (October 2022, 59/77, 77%) and after (June 2023 and October 2023, 45/77, 58%) the program. Of the respondents, 42/77 (55%) had paired pre- and post-survey data. No significant differences existed in the survey between residents who participated in the coaching program and those who were not coached (Table 3).

### Qualitative Results

Sixteen semi-structured interviews provided valuable insights into the mechanisms behind coaching’s influence on SDL. Tables 4, 5 and 6 present the themes within each domain of the IPO framework and provides definitions and exemplar quotations.

**Table 4:** Themes Within the Input Domain of the IPO Framework, Definitions, And Exemplar Quotations

Domain	Themes	Quotes
Input	**Identifying knowledge gaps and structuring learning:** Residents initially struggled with identifying knowledge gaps and structuring self-directed learning. Coaching helped them develop more systematic approaches and better awareness of their learning needs **Influence of coach and coachee attributes:** The attributes of both coaches and coachees influenced the effectiveness of the coaching program. Coaches who were approachable, non-judgmental, and supportive fostered a positive learning climate, while coachees who were open and engaged benefited more from the program	*Prior to the academic coaching program, I had no structure at all. And I said I was intimidated by MKSAP [Medical Knowledge Self-Assessment Program] by having any structure or studying that wasn't for a specific test. That's like how med school was, where you have a test two weeks from now. You have this amount of material to get through. I felt very lost in having this big scary board exam in three years and not knowing how to move through so much material. And so, I think without this academic coaching program, I think I still would have just been wallowing and pretending I knew what I was doing and not having any structure at all.* Resident 16 *…* [SDL] *should be coming from the residents themselves because, ultimately, it's the learner that drives that whole process. So we can tell them they have to do better, but if they don't believe that they can do better or they don't have the desire to be better…I think that that's kind of at the base of it all if they have those things and they just don't have the tools; I think that's where the coach comes in.* Resident 2

**Table 5:** Themes Within the Process Domain of the IPO Framework, Definitions, and Exemplar Quotations

Domain	Themes	Quotes
Process	**Valuing individualized support:** Residents valued the personalized support they received, which included regular feedback and tailored learning strategies. Flexible meeting schedules and virtual options enhanced the program's feasibility **Guidance and customization of learning plans:** Coaches provided critical scaffolding in developing and refining individualized learning plans, helping residents to focus on high-yield areas and develop effective study habits	*… meeting every three months or every month, even if it's just like a phone call or check-in to make sure that we're still on our learning plan. I feel that that's also reasonable. I think the biggest thing for me was when I fell off, it's hard to get back on, so I think frequent checks are needed, especially if they're not going to be long meetings. If you're meeting frequently, you probably don't have to meet for long.* Resident 8 *I definitely think I needed them less as time went on. I think initially, when I was very desperate, desperate is not the right word, but I was very anxious about feeling so lost and feeling. So I was drowning in a sense, and I also had a sense of embarrassment about how lost I was because it seemed like other people were using MKSAP, and other people were already studying, and other people were doing questions. But once we did create a schedule and once we did create that scaffolding, I was able to move through the curriculum on my own and more independently and more meaningfully than I had before when I felt like I was trying, but I wasn't actually doing anything.* Resident 16 *It was very put together and organized. I think that I could have done better to organize it in a way that would have fit with my schedule better,. Still, I feel like it was initially very organized and maybe railed off a little bit until I met with my academic coach and kind of got back on it and then made some adjustments to how I was conducting my individual learning. I had to change my original plan…* Resident 8

**Table 6:** Themes Within the Output Domain of the IPO Framework, Definitions, and Exemplar Quotations

Domain	Themes	Quotes
Output	**Development of metacognition:** Participation in the coaching program increased residents' metacognitive awareness, allowing them to better identify and address their learning gaps. This awareness extended beyond immediate learning goals to broader professional development **Structure and accountability:** The program offered a structured framework that facilitated goal setting, progress tracking, and accountability, which were essential for achieving learning objectives and improving performance on assessments	*I think the concept of everyone's going to have knowledge gaps, and that's okay, but you need to fill those knowledge gaps as much as you can, and then continue to identify areas of improvement and then improving them throughout your career is an important skill to have so that's why I think implementation of an ILP is probably the way that we do it…* Resident 9 *It's the accountability because I would know in the back of my mind those meetings were coming up, and so it served as extra motivation to make progress along, especially on harder rotations.* Resident 7 *…help me stay accountable and stick to the learning objectives. And then, just making sure I kept my motivation up to study that one rough month and I didn't have the time to study … They're talking to you: what is your plan for this month? Then, it keeps you on track even if you fall off the wagon for a month, and it's not hard to find the motivation to get back to your routine.* Resident 12 *I think for the most part, I felt that it gave me confidence, and I felt that it gave me the structure that I needed as well as the accountability that I needed. I think early in my intern year; it was very easy just to kind of hide in my own shadow or, say, I was too busy or kind of ignore the fact that I had responsibilities to myself to learn on my own. And so I think it really, first, gave me accountability and that I needed to create a schedule and needed to create a way that I was going to cover all this curriculum because someone else was relying on me to do that and expected me to create a schedule. And then, outside of that, I definitely think it just helped me think about how I learned and how to create a structure of studying and learning for myself.* Resident 16 *It was a good encouragement to keep going because it's a long process. It's more of a marathon than a short investment. So, I think that the most influential part of it was just the encouragement to keep going and to keep to the plan.* Resident 15

### Input

Residents initially needed help identifying their knowledge gaps and organizing their self-directed learning, which they often described as informal and unstructured before the coaching program. Coaching helped residents develop more systematic approaches and a better awareness of their learning needs. The effectiveness of the coaching program was also influenced by the attributes of both coaches and coachees. Coaches who were approachable, non-judgmental, and supportive fostered a positive learning climate, while coachees who were open and engaged benefited more from the program. Many residents remarked on the value of their relationship with their assigned coach in improving their buy-in and appreciation regarding the program.

### Process

The coaches provided their residents with process-based support focused on direction in one’s learning and “how to” strategies for mitigating knowledge gaps. Residents felt this guidance was essential when veering off track with their learning plans. Coaches suggested a variety of organizational systems and personalized learning strategies tied to their individualized learning plans, and they were flexible with how often to meet with trainees based on their needs. Residents described reflection on their methodological choices during meetings with their coach and would frequently adjust both learning and performance goals. Coaches offered scaffolding or individualized support built upon existing knowledge and structures to improve resident performance.

### Output

The coaching program helped residents develop more awareness, or meta-cognition, related to their knowledge gaps. Coachees were better able to recognize why they had knowledge gaps, how to mitigate them, and how they could continue to use this awareness to drive learning. Some residents could even reflect on their learning behaviors when encountering a patient during an admission or outpatient visit when they had more time on the weekends or after high-stakes summative examinations. The residents learned to self-monitor their learning behaviors and track their results. As they worked through the program, they considered how they would like to study in the future and what their schedule should look like. They felt the program was vital to “learning how I learn” and that the effect of this would transcend beyond residency.

The coaching program provided the structure and accountability necessary to accomplish learning goals and objectives. It offered both internal and external motivations to stick to their learning plans. Motivation was enhanced by their coaching meetings when they were held accountable for their progress on their ILPs. Most residents described a sense of accountability or willingness to accept responsibility for completing their learning objectives. Their scheduled coaching meetings provided the incentive to stay more aligned with their learning plans.

## DISCUSSION

Data from this mixed-methods case study provides insights into the coaching program’s overall effectiveness and a deeper understanding of how the coaching influenced participants. The quantitative findings demonstrate improved IM-ITE scores following coaching program participation. The qualitative data helps to explain these improvements and provides robustness to our findings through complementarity.^24^ Our attitudinal survey revealed no significant differences between the coached and non-coached resident cohorts, and using learning plans alone did not correlate with IM-ITE performance.

We found significant differences in both percent correct and percentile scores on the National IM-ITE between residents who received coaching and those who did not. The effect size was notably greater when the coaching relationship involved two or more meetings, suggesting that sustained coaching positively impacts ITE performance. Although we did not find a prior study explicitly evaluating the impact of coaching on ITE scores, our findings align with Kim’s (2011) systematic review, which identified structured reading and remedial programs as critical predictors of performance on the American Board of Surgery ITE.^25^ Additionally, Chase (2012) found that various factors, including the number of admissions, were associated with ITE performance.^26^ This supports our approach of incorporating active patient care into ILPs, as it offers a practical means for reviewing and addressing knowledge gaps.

Our qualitative findings provide important insights into *how* the coaching program influenced residents’ self-directed learning (SDL). We analyzed the data using an input-process-output (IPO) logic model, a classical framework for organizing and interpreting findings.^23^ In this model, the input includes team members’ skills, knowledge, and attributes.^27^ We identified two key themes within the input domain for our study: many residents initially struggled to identify knowledge gaps and structure their learning going into the program, and attributes and attitudes of the coach and coachee entering the program were instrumental to its success. The process domain links input to output and encompasses task-relevant interactions between team members.^28^ In our study, the ability of coaches to personalize learning plans and provide appropriate scaffolding and support (the process-related themes) was critical to achieving the outputs of improved learner metacognition related to learning and enhanced structure and accountability for completing learning plans.

This analysis aligns with Gallimore and Tharp’s four-step expansion of the ZPD, which emphasizes the role of scaffolding in moving learners from their current abilities to higher levels of understanding.^17^ According to Gallimore and Tharp, effective scaffolding involves a sequence of steps, including modeling, collaborative practice, and independent application. These correspond to our identified themes of guidance and customization of learning plans, and valuing individualized support. While McGrath originally defined output as performance output, this concept has evolved to include behavioral outcomes such as commitment, perceptions of performance, and responses to problems.^23,29^ Our output themes, development of metacognition and structure and accountability, reflect the expansion of the ZPD through the development of self-regulation and increased accountability, underscoring how coaching facilitates growth within the ZPD framework. The importance of scaffolding might also explain why ILPs alone were not found to influence IM-ITE in our trainees; without the structure and accountability to accompany them, the ILPs were not enough to influence performance, but in conjunction with coaching, scores did improve.

The ability to generate meta-inferences from the qualitative and quantitative strands of the study is one of the principal reasons for supporting the value added to a mixed methods study.^30^ Meta-inference of combined data suggests that residents benefit from the coaching program’s scaffolding, yet not all residents perceive changes in their SDL. This lack of insight or awareness contributed to our empirical puzzle. We found no differences between the pre-intervention and post-intervention attitudinal questionnaires in residents who were coached compared to those who were not coached. This result resonates with the findings of Nothnagle (2011), who reported that graduating family medicine residents lacked confidence in their self-directed learning (SDL) skills and sought more external guidance.^31^ In a related cross-sectional analysis, Li (2009) found that junior learners, including residents, often struggle to grasp the concept of SDL, suggesting that they require additional training and experience to understand its importance.^32^ Together, these studies, including ours, highlight a persistent challenge in effectively addressing SDL attitudes among medical trainees. While coaching may not address this perception gap, it underscores the need for comprehensive and structured interventions to build confidence and competence in SDL. For this reason, we propose that some require guided reflection on their SDL skills and that similar academic coaching programs may need to have mandatory enrollment features. This meta-inference aligns with Gallimore and Tharp’s four-stage model of Vygotsky’s ZPD, where tools of scaffolding and support fade as the learner progresses towards automaticity in their SDL.^17^ Our findings illustrate how the coaching program influences residents’ perceptions of their SDL and the impact of this support.

The study has noteworthy limitations. First, it was conducted at a single institution, and its results may need to be more generalizable. Second, we found that 12/40 (30%) of residents enrolled in the coaching program never met with their assigned coach, and their lack of participation could have influenced their attitudinal questionnaire. Third, the cohorts were not randomized and were determined by their enrollment in the coaching program. Fourth, the response rates on our attitudinal questionnaire may affect the reliability of the test results, as responders may differ from nonresponders. Fifth, selection bias may exist, as participants might have higher motivation or stronger SDL skills. Additionally, the timing of the intervention precludes assessment of its long-term impact on American Board of Internal Medicine pass rates. Sixth, a limitation of our study is that our current data infrastructure and availability of historical data do not permit the use of prior cohorts as a control group to adjust for baseline differences in ITE scores. Lastly, our coaching program intervention correlates with improved performance on the IM-ITE, but we acknowledge that other factors aside from the coaching program likely influenced IM-ITE performance. Confounding variables include variations in training experiences, such as differences in clinical rotations, prior medical education or pre-residency SDL training, academic performance, study habits, and test-taking skills. We foresee many areas of future research based on these findings, including an analysis of the number of coach meetings needed to see a meaningful improvement in IM-ITE outcome, the influence of program participation on American Board of Internal Medicine performance, an exploration of burnout with SDL, a deeper understanding of the resources and methods that resident physicians use to promote their SDL, and an analysis of the influence of the individualized learning plan within the coaching program.

## CONCLUSION

Participation in a coaching program within an IM residency correlated with improved performance on the IM-ITE, and this association was strengthened by participation in multiple coaching meetings. Qualitative data suggests that individualization of support, scaffolding, accountability, and structure were key mechanisms by which the coaching program improved SDL among residents.

## Supplementary Information

Below is the link to the electronic supplementary material.

Supplementary file1 (DOCX 4110 KB)

## Data Availability

The datasets generated during and/or analyzed during the current study available from the corresponding author on reasonable request.

## Declarations

### Disclaimer

The views expressed in this work are those of the authors and do not reflect the official policy or position of the Department of the Army, Navy, Air Force, Department of Defense, or the United States Government.
