# Supplementary material for: Academic Coaching to Promote Self-Directed Learning in Graduate Medical Education
Source: J Gen Intern Med. 2025 Feb 13;40(14):3311–9. doi: 10.1007/s11606-025-09424-7 (PMC12586823; doi:10.1007/s11606-025-09424-7)
Supplement: Supplementary file 1 — Supplementary file1 (DOCX 4110 KB) [file 11606_2025_9424_MOESM1_ESM.docx]

Appendix a. Interview Guide

Coaching in Medical Education Resident Interview Script

Thank you for volunteering to participate in this interview. My name is Kathryn Burtson. I am an MHPE candidate at Uniformed Services University. I am interested in the influence of coaching on self-directed learning in graduate medical education. We want to use insights from this interview to inform our program evaluation committee, improve future iterations of the Academic Coaching program, and share this knowledge with the graduate medical education community.

Consent Script: Before starting your interview, I will confirm that you agree to participate. Please answer yes or no to the following questions:

1. Do you agree to participate in the interview today?
2. Do you agree to have this interview recorded and transcribed, knowing all identifying information will be removed during transcription to protect your anonymity?

As a reminder, we will record this interview on Google Transcribe, and I will begin recording now.

Introduction Script: This interview aims to hear your perspectives on your self-directed learning and the academic coaching program. There are many different perceptions of self-directed learning, so we aim to determine what it means to you. As a participant in the coaching program, we desire to discover what motivated you to meet with your coach and how those meetings influenced your self-directed learning.

As we go through the interview today, we want to respect the privacy of the resident physicians and the faculty academic coaches. For this reason, we ask that you not use physician names in the interview. The questions that we will be discussing have no right or wrong answers. We ask that you be honest and open in your responses, but please don’t feel pressured to answer questions that make you uncomfortable. This interview should take less than one hour to complete. If you desire, you may conclude this interview at any time. Do you have any questions before we start this interview?

I have a few questions to start the interview:

1. Did you participate in the Academic Coaching program last year?
2. Please state the year of training you started the Academic Coaching program.
3. How often did you meet with your academic coach?

Thank you. We will now begin with the interview questions.

1. How do you define self-directed learning?

***SDL definition:*** Process by which a motivated learner reflects on their knowledge gaps, develops learning goals, implements learning plans, and evaluates their approaches to learning.

***Probes*:** What does this term mean to you? Do you remember when you first heard this term? Are there any influential people in your past that role-modeled self-directed learning? Did the coaching program influence your definition of self-directed learning?

1. How would you describe your self-directed learning?

***Rephrase, if needed:*** How would you describe your learning outside of the classroom? ***Probes:*** How do you perform a gap assessment between your current knowledge and your ideal state of knowledge? How do you prioritize your learning goals? How do you implement learning plans? How do you monitor your progress with your learning? How do you know you’ve satisfied your goals? Did the coaching program influence the way you perform self-directed learning?

1. How did you use your individualized learning plan?

***Rephrase, if needed*:** How did you use the learning plan you created at a forum with Drs. Burtson and Wilson after the 2022 Internal Medicine In-training Examination?

***Probes:*** Can you describe your individualized learning plan? How did you select your learning goals in your individualized learning plan? What happened after you created your individualized learning plan? Did you satisfy the learning goals written in your individualized learning plan? How did the coaching program influence your individualized learning plan?

1. What motivated you to participate in the coaching program?

***Probes:*** Was your participation voluntary? If so, what drove you to enroll in the coaching program? If your participation was mandatory, were you motivated to participate? Why or why not?

1. How would you describe your experience with coaching in medical education? ***Probes:*** Before the academic coaching program, have you ever had a coach? If so, in what context did you have a coach (sports, music, acting, etc.?) How did that coach differ from your academic coach?
2. How did your meetings with your academic coach differ from those with your resident advisor?

***Probes:*** When you met with your academic coach, what happened? Can you describe a typical meeting with your academic coach? What happened when you met with your resident advisor? Can you describe a typical meeting with your academic advisor?

1. How did the coaching program influence your self-directed learning?

***Probes:*** How would you describe your self-directed learning before making an individualized learning plan and meeting with a coach? How would you describe your self-directed learning while enrolled in the coaching program with an individualized learning plan? How did your coach provide the support/scaffolding necessary to engage in your self-directed? How did that support/scaffolding change/evolve over time (less/same/more?) How would you describe your self-directed learning now that the coaching program is complete for the year?

1. What expectations are reasonable for a resident regarding their self-directed learning? ***Probes:*** How important is it to you to have an individualized learning plan? How important is it to you to have an Academic Coach? What are reasonable expectations of self-directed learning in residents identified as academically at risk of failing their board examinations? Who should monitor a resident's progress with their self-directed learning?

How do you perceive your faculty/attending physician's expectation to engage in self-directed learning?

1. Do you have any personal experiences where you felt you did an excellent job in your self-directed learning?

***Probes:*** If so, what contributed to your performance? How did this experience shape your perception of self-directed learning? What systematic features (such as the coaching program, your individualized learning plan, or your learning goals) could contributed to this performance?

1. Do you have any personal experiences where you did poorly with your self-directed learning?

***Probes:*** If so, what contributed to your performance? How did this experience shape your perception of self-directed learning? What systematic features (such as the coaching program, individualized learning plans, or learning goals) could contribute to this performance?

Summary Questions:

In this last set of questions, I would like to ask you a few closing questions:

1. Before we end the interview, I want to thoroughly cover your thoughts and opinions. Is there anything you want to add that we didn’t discuss?
2. Are you willing to be contacted by a research team member if we have issues with the recording or require clarification on your answers

Appendix b. Individualized Learning Plan Template

| **Individualized Learning Plan** | | |
| --- | --- | --- |
| **Name:** | **Academic Coach:** | |
| **Goals** | | **Options** |
|  | | Medical essay-type questions (MKSAP) |
|  | | Meaningful participation in PBLs |
|  | | Simulated patient encounter |
|  | | Deliver Morning Report |
|  | | Direct patient care IAW EBM |
|  | | Deliver a chalk talk |
| **Objectives** | **Timeline** | **Methods** |
| **Nephrology** |  |  |
|  |  |  |
|  |  |  |
| **Gastroenterology** |  |  |
|  |  |  |
|  |  |  |
| **Gen Internal Medicine** |  |  |
|  |  |  |
|  |  |  |
| **Endocrinology** |  |  |
|  |  |  |
|  |  |  |
| **Hematology/Oncology** |  |  |
|  |  |  |
|  |  |  |
| **Pulmonary Critical Care/Allergy** |  |  |
|  |  |  |
|  |  |  |
| **Cardiology** |  |  |
|  |  |  |
|  |  |  |
| **Neurology** |  |  |
|  |  |  |
|  |  |  |
| **Women's Health/Dermatology** |  |  |
|  |  |  |
|  |  |  |
| **Infectious Diseases** |  |  |
|  |  |  |
|  |  |  |
| **Geriatric Medicine** |  |  |
|  |  |  |
|  |  |  |

Appendix c. Initial Coach Script for Faculty


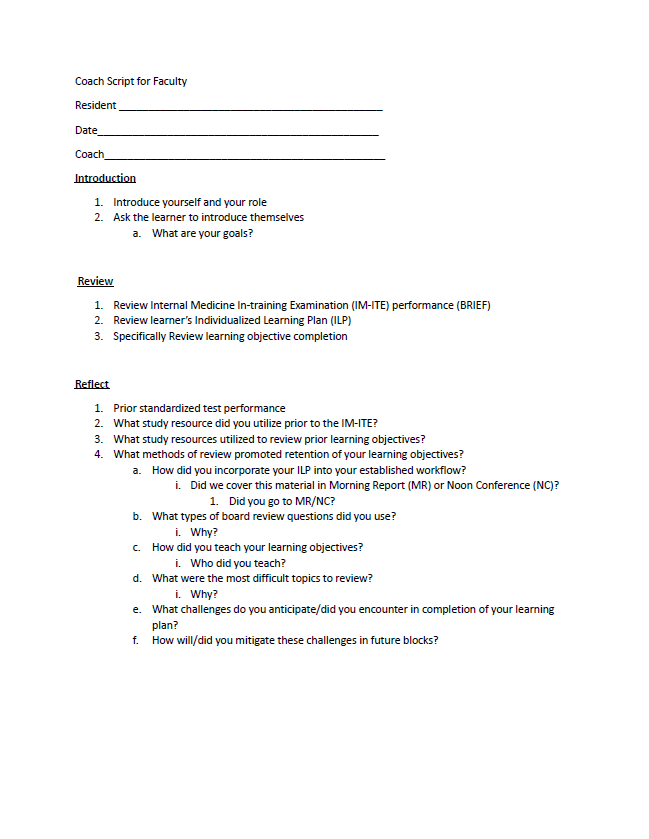


Appendix d. Coach Script for Faculty Subsequent Encounter


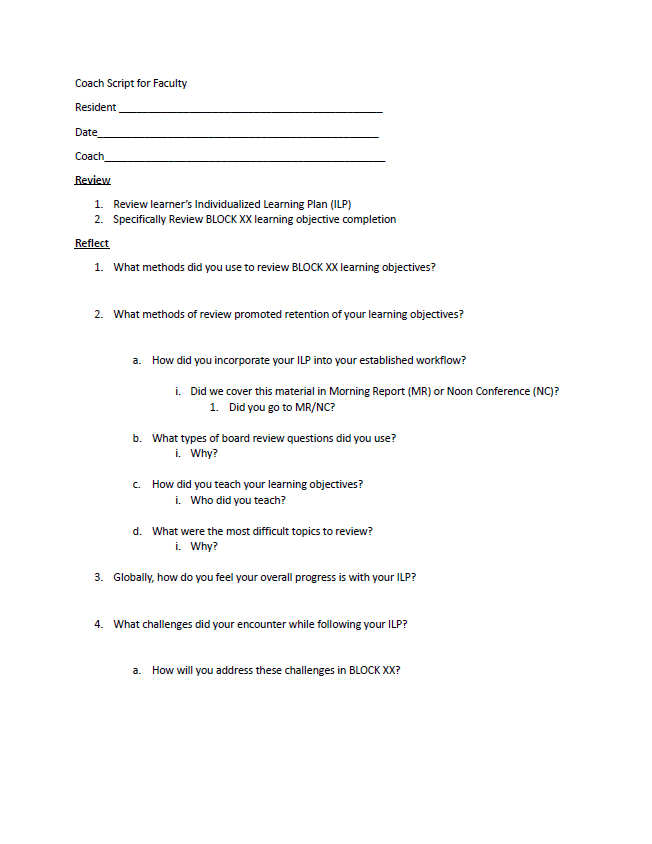


Appendix e. Coaching Contract

**
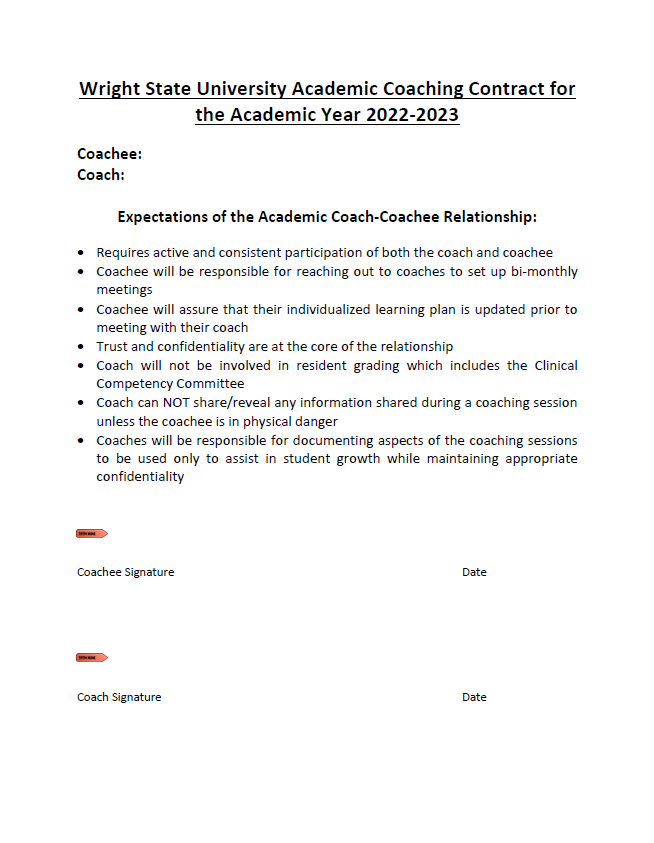
**
